# Influence of latrine coverage and usage on diarrhoea incidence among children under 5 living in slum areas of Douala 5^th^ sub-division, Cameroon

**DOI:** 10.11604/pamj.2023.44.82.11996

**Published:** 2023-02-09

**Authors:** Rodrigue Biguioh Mabvouna, Adogaye Sali Ben Béchir, Martial Patrick Pete Nkamedjie, Vittorio Colizzi, Martin Sobze Sanou

**Affiliations:** 1 Faculty of Medicine and Surgery, University of Roma “Tor Vergata”, Roma, Italy,; 2 Institute for Research, Socio-economic Development and Communication (IRESCO), Yaoundé, Cameroon,; 3 Department of Biomedical Sciences, Faculty of Sciences, University of Dschang, Dschang, Cameroon

**Keywords:** Diarrhoea incidence, latrine coverage, children under 5, slum areas

## Abstract

**Introduction:**

lack of adequate sanitation facilities remain a major concern in developing countries. While around 41% of Cameroonians lack access to improved sanitation facilities, the 2011 National Survey revealed a diarrhoea incidence rate of 21% in children under five years, two weeks before interview. This study aimed at evaluating the influence of latrine coverage and usage on diarrhoeic disease outcomes among children under 5.

**Methods:**

a cross-sectional study was carried out in March 2016 in pre-selected slums areas of Douala 5^th^ district. A structured questionnaire was used to collect data from one consenting adult per household. Data analysis was carried out using Epi Info version 7.1.4.0. Pearson’s chi-square and Fisher exact test were used to estimate the influence of latrine coverage on the incidence of diarrhoea. Statistical significance was set at p < 0.05.

**Results:**

of the 384 households enrolled, 69.01% had latrine facilities, while 30.99% shared latrines with neighbouring households. Sixty point sixteen percent (60.16%) (231/384) of all households used pit latrines. Although consistent use of latrines by all adults was reported, 20.05% of children under 5 practiced open-air defecation. The incidence of diarrhoea among children under 5 years 2 weeks before interview was 29.25%, of which 26.35% were bloody stools. Diarrhoea outcome was significantly associated with use of pit latrines (p < 0.01); lack of cover on latrines hole (p < 0.0001) and proximity of latrines to household (p=0.01).

**Conclusion:**

poor faecal waste management and lack of improved sanitation facilities contribute significantly to diarrhoeal episodes among children under 5. A structured strategy to improve community-based sanitation considering urban planning and sanitation campaigns would promote safer environment and reduce outcome of water-borne and diarrhoeic diseases.

## Introduction

Sanitation refers to facilities and services for collecting and managing excreta and community liquid wastes hygienically to help preserve the health of individuals and that of the community as a whole [1]. Approximatively, 2.5 billion people in the world lack access to improved sanitation facilities such as a basic pit latrine for the proper disposal of human excreta [2]. In Cameroon, since the early 2000s, considerable progress has been made in terms of safe drinking water, hygiene and sanitation [3-7]. However, huge efforts are still required regarding access to improved sanitation [8-14]. According to Cameroonian Demographic Health Surveys (DHS), the estimated proportion of the population without access to improved sanitation facilities was 70% in 1998 [9], 64% in 2000 [10], 56% in 2004 [11], and 41% in 2011 [12]. There is still a huge gap between urban and rural areas, with 67% and 15% of the rural and urban populations respectively lacking access to improved sanitation facilities [12,13]. Moreover, the latest data reveals that 7.2% of Cameroonian households have no sanitation facilities [12,13]. Among those with sanitation, 33.2% use pit latrines without slab or a secure hole, 23.3% share sanitation facilities with other households (of which 21.2% are pit latrines without slab) [12,13].

Diarrhoea alone kills more children than AIDS, malaria, and measles all combined [14]. Latest DHS of 2011 indicates that diarrhoeal prevalence two weeks prior investigation in under 5 children in Cameroon is about 21% [12]. Other surveys conducted in Cameroon revealed a diarrhoea prevalence of 23.8% [15], mostly associated with poor sanitation and unsafe water [16]. Diarrhoea is the second childhood diseases after malaria and accounts for about 23% of outpatient consultations in Cameroon [12]. Diarrhoea causes malnutrition and dehydration in children due to the loss of water in faeces, resulting in children being highly susceptible to diseases [14]. Douala is a coastal town with tropical climate and abundant rainfall often resulting in frequent flooding due to lack of wastewater and rainwater management systems. Thus, the town is prone to frequent diarrhoeic diseases outbreaks, including cholera [17-22] resulting from contamination of drinking water by faeces [17,20,22]. Open-air defecation and the use of unimproved latrines have been observed in some areas of the town, posing a serious public health concern [12,16]. Moreover, considering the economic situation [12,13], Douala is the destination for great number of people migrating from rural areas in search of employment. This leads to over population and the creation of slums characterized by poor sanitation as is the case in Douala 5^th^ sub-division [22-25]. The aim of this study was to assess the influence of latrine coverage on the incidence of diarrhoeic diseases among children under 5.

## Methods

**Study design and period:** a community-based cross-sectional study was conducted among population living in slum areas of Douala 5^th^ Sub-division. Data were collected in March 2016.

**Study area and population:** Douala 5^th^ is a municipality sub-division of the urban community of Douala, Wouri department in the Littoral region of Cameroon. Douala 5^th^ is both urban and rural and has an estimated population of 544,919 inhabitants [5]. The spoken local language is Douala, which shares its name with the city. Although Douala is the economic capital of Cameroon, it also faces some problems like other underdeveloped countries such as lack of employment, criminality, promiscuity, recurrent diseases outbreaks, lack of potable water and bad climate (heavy rain, flood). A large percentage of Douala population live below the poverty line (about 35%). Poverty is a growing problem for Douala due to its steadily increasing population, and the inhabitants do not have many opportunities for monetary gain.

**Sample size and sampling:** the study sample size was calculated using a single population proportion formula, assuming that 50% of the local population have improved latrines, with a precision of 5%, an alpha error of 0.05 (confidence level of 95%), which resulted in 384 households to be included. Nine slums were randomly selected from a list provided by Douala 5th sub-division authorities with probability proportional to their population size. The number of households to include per slums was obtained based on the estimated population in each slum. Due to the unavailability of the exhaustive list of households in each sampled slum, the sampling of households was done starting from the geographic centre of the slum. Using the spinning bottle method, the household directions for data collection were randomly determined and all households in each direction were visited. Households with at least one child under 5 were recruited consecutively until the planned slum sample size was reached.

**Data collection:** a structured interviewer-administered questionnaire was used to collect data from one consenting person per household, preferably the head of the family. To determine the incidence of diarrhoeic diseases, respondents were asked if at least one child under 5 in the household suffered diarrhoea (≥ 3 stools per day) during the two weeks preceding data collection.

**Data analysis:** statistical analysis was carried out using Epi Info version 7.1.4.0 and descriptive statistics were reported. Pearson’s Chi-square and Fisher’s exact test were used to estimate the influence of latrine coverage on occurrence of diarrhoea. Statistical significance was set at p < 0.05.

**Ethical considerations:** all consenting respondents were adults and the questionnaires were administered only after obtaining the informed consent. No personal information was collected, and each questionnaire was given a unique identification number. All necessary information regarding the study description were provided to participants. We explained the objectives of the study, data collection methodology, possible discomforts, risks and benefits. We also explained the participants' right to withdraw from the study before and during the study without any prejudices.

## Results

### Socio-demographic characteristics of the study population

Overall, 384 households from 9 slums of Douala 5^th^ sub-division were enrolled in the study (Table 1). Respondents were either the father or the mother of the family, mainly living in private houses (59.64%). Regarding the family size, 74.22% were families of < 5 individuals, and 1 family out of 4 (28.13%) had ≥ 2 children under 5. Overall, 506 children under 5, mostly aged 12 - 23 months (23.1%) of male sex (55.7%) were investigated in the households visited (Table 2).

**Table 1:** distribution of samples by slum areas

Slum	Number of households surveyed	Number of children surveyed
Beedi	39	46
Bépanda	50	75
Cité des palmiers	52	98
Makepé Missoké	50	65
Ndog-bong	39	43
PK 8	44	55
PK 10	40	45
PK 9	33	39
PK 14	37	40
**Total**	**384**	**506**

**Table 2:** sociodemographic characteristics of households and children included in the study

	Frequency	Percentage (%)
**Gender of head of household**		
Male	91	23.7
Female	293	76.3
**Type of habitation**		
Private	229	59.64
Cohabitation	155	40.36
**Family size**		
< 5 persons	285	74.22
≥ 5 persons	99	25.78
**Number of children under 5 by sex**		
Female	224	44.3
Male	282	55.7
**Age of children (in months)**		
<6	82	16.2
6 – 11	95	18.8
12 – 23	117	23.1
24 – 35	88	17.4
36 – 47	74	14.6
48 – 59	50	9.9

### Latrine coverage, description and utilization

Table 3 summarises the characteristics of the latrines observed. Sixty-nine point one percent (69.01%) of households had latrine facilities, 30.99% shared latrines with neighbouring houses. All the latrines were also used as bathrooms, and up to 60.16% (n=231) of all latrines were pit latrines, others were flush toilets (39.84%). Among the flush toilets (n=153), 44.44% were equipped with a functional water supply installation (cistern or holding tank) and 34.64% had a hand-washing facility. On the other hand, all the pit latrines observed during the survey had no hand-washing facilities and only 16.45% had a covering device for the hole. While 36.72% of latrines were located inside the house, 63.28% were found outside (55.56% of them at < 6 meters of distance from the house). Concerning the building materials of external latrine facilities, 70.78% had a cemented (slab) floor, 39.51% possessed a roof in metal sheets, and 38.68% had walls made up of wood planks. Regarding the utilisation of latrines, consistent use by all adults was reported by respondents. 20.05% of respondents (n=384) admitted open-air defecation for children under 5, and 65.89% used the latrines directly or indirectly (14.06%) by collecting faeces in potty which are then emptied in latrines. However, it appears that 54.95% of respondents consider their latrines to be a serious health hazard (Table 4).

**Table 3:** description of latrines used by households

	Frequency	Percentage (%)
**Latrine category**		
Private	265	69.01
Shared	119	30.99
**Type of latrine**		
Water-based toilet (flush toilet)	153	39.84
Pit latrine	231	60.16
**Water supply installation (only for water-based toilets)**		
Yes	68	44.44
No	85	55.56
**Hand washing facility**		
Yes	53	13.8
No	331	86.2
**Pit latrine covered**		
Yes	38	16.45
No	193	83.55
**Location of the latrine**		
Inside the house	141	36.72
Outside the house	243	63.28
**Distance between latrine and house (only for external latrines)**		
< 6 meters	135	55.56
6 – 10 meters	108	44.44

**Table 4:** place of defecation for children under 5 and opinion on latrine

	Frequency	Percentage (%)
**Place where children under 5 defecate**		
Latrine	253	65.89
Potty and emptied into latrine	54	14.06
Open air defecation	77	20.05
**Opinion on latrine**		
Good quality	173	45.05
Present a health risk	211	54.95

### Diarrhoea incidence among children under 5

As shown in Table 5, 29.25% (n=148) of children under 5 included in the study experienced at least one episode of diarrhoea two weeks before the survey of which 26.35% were bloody stools. No variation of diarrhoea incidence in relation to the place of residence was found (p-value = 0.39). Cases of diarrhoea were mostly recorded in slums of Bépanda and Makepé Missoké (14.2%). Episodes of diarrhoea with bloody stools were mostly noted in slum of Cité des Palmiers (17.9%). The incidence of diarrhoea was higher among children aged 12 - 23 months with 31.8% (n=47) of diarrhoea cases recorded in this age group. Children of male sex seem to be most affected accounting for 56.1% of diarrhoea cases observed. However, overall distribution of diarrhoea cases according to sex revealed that males and females were similarly affected: 29.08% (n=65) for females and 29.43% (n=83) for males. No significant association was found between the occurrence of diarrhoea and age (p-value = 0.06) or sex of children (p-value = 0.07). However, highlight significant association between incidence of diarrhoea and the use of pit latrines (p-value = 0.00). Indeed, 68.9% of diarrhoea cases were recorded in households using pit latrines against 31.1% in households with water-based toilet (flush toilet). Moreover, 44.2% of households using pit latrines experienced at least one episode of diarrhoea during the two weeks preceding the survey compared to 30.1% in households with water-based toilet (flush toilet). Similarly, bloody stools were mostly present in households using pit latrines; 66.7% of cases against 33.3% in households with water-based toilet (flush toilet), but the difference was not significant (p-value = 0.38).

**Table 5:** incidence of diarrhoea according to age, sex, type of latrines and children’s place of defecation

	All types of diarrhoea (N=148)		Bloody diarrhoea (N=39)	
	Frequency	Percentage (%)	Frequency	Percentage (%)
**Age of children in months**				
<6	22	14.9	5	12.8
6 – 11	20	13.5	6	15.4
12 – 23	47	31.8	14	35.9
24 – 35	26	17.6	10	25.6
36 – 47	18	12.2	3	7.7
48 – 59	15	10.1	1	2.6
**Sex of children**				
Female	65	43.9	16	41
Male	83	56.1	23	59
**Type of latrine**				
Water-based toilet (flush toilet)	46	31.1	13	33.3
Pit latrine	102	68.9	26	66.7
**Pit latrine with covering device**				
Yes	19	18.6	5	19.2
No	83	81.4	21	80.8
**Location of the latrine**				
Inside the house	43	29.1	12	30.8
Outside the house	105	70.9	27	69.2
**Distance between external latrine and house**				
< 6 meters	60	57.1	16	59.3
6 – 10 meters	45	42.9	11	40.7
**Place where children < 5 years defecate**				
Latrine	105	70.9	28	71.8
Potty and emptied into latrine	13	8.8	3	7.7
Open air defecation	30	20.3	8	20.5

This study found a significant association between the lack of cover on pit latrines holes and the occurrence of diarrhoea among children (p-value < 0.000). Among the 102 cases of diarrhoea recorded in households using pit latrines, 81.4% were recorded in households using uncovered pit latrines, while only 18.6% were found in households with covered pit latrines. Furthermore, of the 26 cases of diarrhoea with bloody stools recorded in households with pit latrines, 80.8% were observed in households with uncovered pit latrines, against 19.2% of cases in households with covered pit latrines (p-value =0.02). A significant association was also found between the incidence of diarrhoea and latrine´s proximity with living space (p-value =0.01). Most cases of diarrhoea (70.9%) were observed in households with external latrines, and among them, 57.1% in households with latrines located at a distance < 6 meters from the living space. Finally, 20.3% of diarrhoea cases were observed in households where children practiced open air defecation, corresponding to a diarrhoea incidence of 39% in those households.

**Limitations:** diarrhoeic diseases and their consequences (malnutrition and dehydration), are either directly or indirectly the second leading causes of death among children under 5 in developing countries [26-28]. Despite some limitations, mainly lack of assessment of household income, parent´s educational level and their knowledge on hygiene rules, findings from this study outline the importance of latrine and faecal waste management in reducing the consequences of diarrhoea among children.

## Discussion

### Latrine coverage, description and utilization

Except from the expanded program of immunization, most health indicators including sustainable access to improved sanitation and safe drinking water have not been significantly improved since 1990 in Cameroon [12,13]. In this study, 30.99% of households lacked private latrines, 60.16% of latrines were pit latrines and 63.28% were external latrines. Among external latrines, 29.22% had floors in wood planks, 60.49% possessed no form of roofing and only 26.75% of latrines had walls built with concrete. In addition, only 16.45% of pit latrines had a covering device that could adequately fit the toilet hole. These results are similar with findings of the Cameroon DHS carried out in 2011, which indicated that about 61.9% of the population used unimproved latrines [12]. These results are also consistent with those of other authors, who suggested that pit latrines are mainly used in low-income settings because of their low cost and availability [29,30]. However, the extensive use of pit latrines could have impacts on human health due to faecal contamination of groundwater and surface water during flooding.

Indeed, because of the low coverage of drinking water supply system and frequent water shortage, local authorities and some inhabitants have undertaken water drilling construction to meet their needs. Nevertheless, pit latrines generally lack a physical barrier, such as concrete between stored excreta and soil and/or groundwater [31]. As such, contaminants from pit latrines may potentially leak into groundwater, making drilling water potentially risky for human health. Beyond health impact and air pollution (disagreeable odour), pit latrines also have an economic impact because they are single use age and the family must build another one once the pit is full, this implies new financial investment. While the 4^th^ Cameroonian DHS indicated that 21% of children under 5 suffered from diarrhoea [12], studies highlight that handwashing with soap reduces the risk of diarrhoea by 48% [30,32]. In this study, 55.56% of water-base toilets had no functional water-supply installation and 65.36% had no hand-washing facilities. Moreover, all the pit latrines had no form of hand-washing facilities and only 16.45% of them had a suitable cover device. These findings can partly explain the high incidence of faeco-oral waterborne diseases in slum dwellers especially among young children [16,22].

Despite WHO recommendations to build latrines with minimum distance of 6 meters away from the living environment in order to avoid the associated health risks [29,30,33], 55.56% of external latrines were located less than 6 meters away from the households. Open air defecation by children was reported by 20.05% of respondents. Respondents explained that open air defecation is more comfortable and better than using latrine for young children. We already known that cholera is associated with low income settings and poor sanitation [33-35], but practices and attitudes of the respondents regarding environmental hygiene can also explain this relation. Indeed, about 54.95% of respondents declared that their latrine presented a health risk for users.

### Influence of latrines on diarrhoea incidence among children under 5 and latrine influence

In this study, diarrhoea incidence two weeks before investigation among children under 5 was found to be 29.25%. This incidence of diarrhoea among children under 5 differs from that obtained from the Cameroon DHS (21%) [12], and the study carried out in Tiko, South-ouest region of Cameroon (23.8%) [15]. It also differs as compared to the study carried in Ghana, Nepal and Egypt which revealed 18% [36], 22.5% [37], and 23.6% [38] respectively. However, this study finding was almost similar to the incidence (28.9%) obtained by survey carried out in Nekemte town, Western of Ethiopia [39]. These variations may be due to the differences in sample size, data collection methods and study sites. Limited access to safe water and improved sanitation in study sites (slums) could also explain these differences. Children aged 12 - 23 months were most affected by diarrhoea (31.8%). This age group correspond to the period during which children start to receive other food supplements in addition to breast milk [14]. This age group also corresponds with moment that children begin to explore their environment, exposing them further to diarrhoea illness through ingestion of contaminated water by faeces which results from poor sanitation [33,40,41].

On the other way, our findings suggest an association between the incidence of diarrhoea among children and the use of pit latrines; diarrhoea incidence found in households using pit latrines was two times greater than the incidence recorded in households with water-based toilets. As already established by other authors, faeces disposal is an important factor in the dissemination of waterborne diseases causing agents [40,41]. Most pit latrines observed in this study were approximately 1-meter depth whose contents are emptied into small drains or rivers via an evacuation pipe during rains. Preference and consumption of spring water referred to as “free water” and perceived as “pure” by local populations compared to tap water called “trapped water” had already been identified as a major determinant of cholera in Douala [16].

Utilization of improved latrines built following standards and located at recommended distance from living space considerably reduce diarrhoea risk [33,42,43]. In the present study, among the 102 cases of diarrhoea observed in households using pit latrines, 81.4% were identified in households using uncovered pit latrines. Also, the number of diarrhoeal cases in households with external latrines located at < 6 meters from the households (57.1%) was high. Nonetheless, diarrhoea outcome was not associated with these latrines´ characteristics. This assertion is in line with a study conducted in Ethiopia which revealed no association between diarrhoea incidence and the location of latrines or the presence of covering device on the latrines [44]. Despite latrine presence, 20.05% of households reported open air defecation for children with 20.3% diarrhoea cases observed among them. Respondents mentioned the convenience of open-air defecation for children. Similar results were observed in another study conducted in Maharashtra (India) [45]. “Open air defecation better“““” (17.4%) than defecate in latrines, was cited among other reasons that justify this practice.

## Conclusion

Despite the availability of latrines in households included in the present study, over half of them are unimproved latrines and they lack adequate sanitation facilities (hand-washing, water installation, suitable covering device). Half of external latrines did not respect standard recommendations representing a serious health risk for the neighbouring populations. Some children practice open air defecation. This practice can lead to groundwater contamination and an increased risk of diarrhoeic diseases transmission such as cholera. In addition to financial expenses, inadequate water supply and lack access to safe water in houses are factors that could also explain low utilization of water-base toilets (flush toilet) and high diarrhoea incidence among children. Operational planning and implementation of sanitation campaigns, communication for behavioural change, and increased access to safe water are cornerstones in improving the current situation.

### What is known about this topic

- *Faecal contamination of water accounts for many water-borne and diarrhoeic diseases such as cholera;*
- *Previous studies revealed an association between diarrhoea outbreak, poor sanitation and unsafe water;*
- *Strict application of hygienic rules contributes in significant reduction of diarrhoea incidence by limiting ingestion of contaminated water or foods by faeces*.

### What this study adds

- *Pit latrines are common sanitation facilities used in slum areas of Douala 5^th^ sub-division;*
- *Poor access to improved sanitation combined with lack of water facilities have a detrimental impact on the health of children under 5, as seen through the high incidence of diarrhoea;*
- *Some households shared latrines with neighbouring houses, but those latrines are hardly used, and people are preferring practice open defecation mostly children under 5*.
